# 5-Hydroxytryptophan Suppresses the Abdominal Fat Deposit and Is Beneficial to the Intestinal Immune Function in Broilers

**DOI:** 10.3389/fphys.2020.00655

**Published:** 2020-06-12

**Authors:** Hui Wang, Shaoqiong Liu, Jun Li, Liyuan Wang, Xiaojuan Wang, Jingpeng Zhao, Hongchao Jiao, Hai Lin

**Affiliations:** ^1^Shandong Provincial Key Laboratory of Animal Biotechnology and Disease Control and Prevention, Shandong Agricultural University, Tai’an, China; ^2^Key Laboratory of Etiology and Epidemiology of Emerging Infectious Diseases in Universities of Shandong, Shandong First Medical University and Shandong Academy of Medical Sciences, Tai’an, China

**Keywords:** 5-hydroxytryptophan, lipid metabolism, intestinal inflammation, LPS, broiler

## Abstract

**Background:**

Serotonin (5-HT), a monoaminergic neurotransmitter, involves in the regulation of many physiological functions. In the present study, the effects of 5-hydroxytryptophan (5-HTP), the precursor of 5-HT, on lipid metabolism and intestinal immune function in broiler chickens were investigated in chickens.

**Methods:**

Two hundred broilers were divided randomly into two groups and fed separately with a corn-soybean basal diet (CD) or the basal diet supplemented with 0.2% 5-HTP.

**Results:**

The results showed that 5-HTP reduced (*P* < 0.05) feed intake and the abdominal fat pad weight. 5-HTP treatment tended to upregulate the mRNA level of adiponectin receptor 1 (ADP1R) and ADP2R in abdominal fat but had no significant influence on their protein levels (*P* > 0.05). In 5-HTP-chickens, lipopolysaccharide exposure decreased secretory immunoglobulin A (sIgA) concentrations in serum and the duodenal contents. Expression of mRNA encoding interleukin (IL), tumor necrosis factor-α (TNF-α), and transforming growth factor-β (TGF-β) decreased after 5-HTP treatment; however, LPS increased expression significantly in 5-HTP-treated chickens compared with CD chickens. In 5-HTP-chickens, the phosphorylation of mitogen-activated protein kinase (MAPK) and nuclear factor-kappa B (NF-κB) were reduced, but the phosphorylation of ribosomal p70S6 kinase (p70S6K) was increased in the duodenum.

**Conclusion:**

In summary, the result suggests that dietary 5-HTP supplementation reduces accumulation of abdominal fat and is beneficial to intestinal immune function.

## Introduction

Serotonin (5-HT) is a monoaminergic neurotransmitter that modulates the function of the central and peripheral nervous systems (Veenstra-VanderWeele et al., 2000). There are two independent 5-HT systems: the central nervous system and the peripheral system. Central 5-HT is synthesized by tryptophan hydroxylase 2 (TPH2), the rate-limiting enzyme for 5-HT biosynthesis (Walther et al., 2003). Peripheral 5-HT is synthesized by intestinal enterochromaffin (EC) cells upon the catalysis by tryptophan hydroxylase 1 (TPH1; Sun et al., 2019). As 5-HT is unable to cross the blood–brain barrier (El-Merahbi et al., 2015) the lack of peripheral or central 5-HT cannot be replaced by the other resource. Once released, 5-HT combines with 5-HT receptors, most of which are G-protein-coupled receptors (except receptor 3, which is a ligand-gated cation channel) (Oh et al., 2015). In chicken and other species, the CNS and peripheral serotonergic systems act independently and meanwhile interact via the blood stream, as affected by hormones (corticosterone, cytokines, enzymes, and short-chain fatty acids), gut microbiota, and vagus nerve activation (de Haas and van der Eijk, 2018).

In rats, peripheral 5-HT changes lipid metabolism by accelerating bile acid turnover (Watanabe et al., 2010). In mice, 5-HT can induce lipogenesis or triglyceride (TG) synthesis in the liver and white adipose tissue (Namkung et al., 2015). Inhibiting peripheral 5-HT synthesis reduces obesity and induces metabolic dysfunction by promoting brown adipose tissue thermogenesis (Crane et al., 2015). In poultry, however, lipid metabolism and its regulatory mechanism are different from those in mammals. The main site of lipid synthesis is the liver and the lipogenesis in adipose tissues is limited (Brady et al., 1976; Bedu et al., 2002). Thus, the effect of 5-HT on lipid metabolism in chickens remains to be elucidated.

The peripheral 5-HT is synthesized in EC cells within the gastrointestinal mucosa and therefore the role of 5-HT on gut immunity gains more attention. 5-HT is an important mucosal signaling molecule, playing vital roles in inflammatory conditions of the gut (Ghia et al., 2009). There is increasing evidence supports the concept that 5-HT is directly involved in pathological mechanisms, as well as the modulation of immune/inflammatory responses within the gut (Cirillo et al., 2011). 5-HT could enhance the host immune function by inhibiting the production of TNF-α (Perianayagam et al., 2005). Recently, the distinct correlation between 5-HT levels and the occurrence of stress-induced diarrhea was observed in weaning mice, which may result in the deregulation of the mucosal immune system (Dong et al., 2017). In broiler, L-tryptophan supplementation could relieve stress-effected expression of IL-1, IL-6, IL-10, and TNF-α by regulating 5-HT metabolism (Yue et al., 2017) and increase serum IgM concentration quadratically in laying hen reared under hot and humid summer conditions (Dong et al., 2012). Except of the behavioral responses, early-life microbiota transplantation has long-term effects on immune characteristics and peripheral serotonin of chicken in a genotype-dependent way (van der Eijk et al., 2020). As the intermediate metabolite of the essential amino acid L-tryptophan in the biosynthesis of 5-HT, 5-Hydroxytryptophan (5-HTP) has been used as a clinically effective serotonin precursor (Birdsall, 1998). 5-HTP is present at low levels in the nervous system because it is rapidly converted to 5-HT (Sloley and Juorio, 1995). 5-HTP is therapeutically used for several psychiatric disorders such as anxiety and depression in the clinic (Murray, 2000; Sahelian, 2000). In broilers, it was reported that there is a possible interaction of 5-HTP and *Lactobacillus* spp. on the immune system of broiler chickens (Donato et al., 2015). Therefore, we hypothesized that 5-HTP may play a role in intestinal immune function in chickens.

Here, we examined the effect of dietary supplementation of 5-HTP on lipid metabolism and intestinal immune function. The transcriptional level of genes related to lipid synthesis in the liver and abdominal fat pad was determined. The concentrations of immunoglobulin A (IgA) and mRNA level of cytokines in the intestinal tract were measured to evaluate the immune function of lipopolysaccharide (LPS)-challenged chickens.

## Materials and Methods

The study was approved by Ethics Committee of the Shandong Agricultural University and carried out in accordance with the “Regulations for the Administration of Affairs Concerning Experimental Animals Guidelines” and “Guiding Directive s for Humane Treatment of Laboratory Animals” by the Ministry of Science and Technology (1988a, b). All husbandry practices and euthanasia were performed with full consideration of animal welfare.

### Animal Management and Sample Collection

Two hundred day-old male broilers (Arbor Acres) were obtained from a local hatching farm (Dabao, Taian, China). All the chicks were reared in an environmentally controlled room, and the rearing temperature was maintained at 33°C from Day 1 to Day 5 and then gradually reduced, according to normal management practices, to a temperature of 22°C. Chickens were reared in floor pens (2 m × 2 m) using rice hulls as litter. Each pen was equipped with tube feeders (the feeder space per bird was 2.5 cm) and nipple water-line (12 birds per nipple). The light regimen was 23 L:1 D and the dark period was from 0:00 to 01:00 a.m. (Zhao et al., 2012; Tang et al., 2019).

The experiment started from the Day 8 to let all the broiler chicks adapt the rearing environment (Yassin et al., 2009; Yerpes et al., 2020). All the experimental chicks were fed with a corn-soybean basal diet (21% crude protein) until 7 days of age. Thereafter, the chicks were assigned to eight pens of 25 chicks according to body weight. The eight pens of broilers were randomly divided into two groups composed of four pen each and subjected to one of the two following treatments: fed with the basal diet (CD), or the basal diet supplemented with 0.2 kg 5-HTP per 100 kg diet (Donato et al., 2015). The basal diets were formulated according to the growing stage of broilers: the starter diet with 21% crude protein and 12.6 MJ/kg metabolizable energy (ME) was fed from 1 to 21 days of age; the grower diet with 19% crude protein and 13.0 MJ ME/kg was fed from 22 to the end of experiment (Table 1; Zhao et al., 2012; Chen et al., 2018). All the broilers had free access to feed and water during the entire experimental period. Body weight (BW) and feed intake (FI) were recorded weekly and the feed conversion ratio (FCR) were calculated.

**TABLE 1:** The composition and nutrient levels of the experimental diets.

Ingredients (%)	1–21 days	21–35 days
Corn (8.5% CP)	57.00	62.00
Soybean meal (46% CP)	34.40	29.50
Soya bean oil	3.90	4.50
Dicalcium phosphate	1.70	1.55
L-Limestone	1.67	1.42
Sodium chloride	0.31	0.28
Choline chloride (60%)	0.26	0.20
L-Lysine⋅H_2_SO_4_ (70%)	0.02	–
DL-methionine (98.5%)	0.49	0.30
Vitamin premix*	0.05	0.05
Mineral premix^†^	0.20	0.20
Total	100	100
**Calculated chemical composition**		
CP, %	21.0	19.0
ME (MJ/kg)	12.6	13.0
Ca, %	0.94	0.90
Available P, %	0.43	0.40
Lys, %	1.10	0.97
TSAA, %	0.87	0.66
Thr, %	0.77	0.70
Trp, %	0.26	0.23
Val, %	0.86	0.78
Ile, %	0.87	0.78
Leu, %	1.60	1.52
Phe, %	1.08	0.98
Arg, %	1.39	1.24
His, %	0.56	0.51
Gly, %	0.82	0.74
Gly + Ser, %	1.78	1.62

*Vitamin premix provides the following per kg of diet: VA, 8000 IU; VD_3_, 3000 IU; VE, 20 IU; VK, 2 mg; VB1, 4 mg; riboflavin, 8 mg; D-pantothenic acid, 11 mg; VB5, 40 mg; VB6, 4 mg; VB12, 0.02 mg; biotin, 0.15 mg; folic acid, 1.0 mg; choline, 700 mg. ^†^Mineral premix provides the following per kg of diet: Fe (as ferrous sulfate), 80 mg; Zn (as zinc sulfate), 75 mg; Mn (as manganese sulfate), 80 mg; Cu (as copper sulfate) 10 mg, I (as potassium iodide), 0.40 mg; and Se (as sodium selenite), 0.30 mg.

After 35-day of age, two broilers around mean body weight were randomly selected from each pen, eight broilers in total in each treatment. After overnight feed withdrawal, a blood sample was drawn from a wing vein with a syringe from each chicken. The blood sample was, respectively, collected with the ice-cold normal and heparinized tube. The plasma or serum was obtained by centrifugation at 400 *g* at 4°C for 10 min and stored at −20°C for further analysis. Immediately after the blood sample was obtained, broilers were sacrificed by exsanguination after cervical dislocation (Close et al., 1997; Huang et al., 2015). Liver and abdominal fat pad (fat in abdominal area) were weighed and sampled. All tissue samples were snap-frozen in liquid nitrogen and stored at −80°C prior to further analysis.

### LPS Treatment

At 35 days of age, four broilers from each pen and 16 chickens in total in each treatment were selected. In each treatment, half of the chickens were received an intraperitoneal injection of LPS (1 mg/kg of body weight, derived from *Escherichia coli* 055:B5, L2880; Sigma-Aldrich, United States) according to Liu et al. (2016) the other half received a sham injection with saline. During the 4-h period of LPS exposure, feed was withdrawn and the broilers had free access to water. At 4 h post-injection, all chickens were sacrificed as forementioned above and the duodenum, jejunum, and ileum were sampled, snap-frozen in liquid nitrogen, and stored at −80°C for the analysis of gene expression. The midpoint of duodenum, jejunum, and ileum were obtained and flushed with 5 mL of ice-cold PBS supplemented with protease inhibitor cocktail according to Burns et al. (2018). The obtained intestinal content was vortexed for 5 min and then centrifuged at 4°C for 30 min, and then the supernatant was collected and stored at −80°C for the measurement of sIgA.

### Measurement of sIgA Concentrations

Immunoassay plates (Costar, Corning) were coated with 100 μL of goat anti-chicken IgA (A30-103A; Bethyl) dissolved in phosphate buffer solution (PBS, pH 9.6) and incubated for 1 h at 37°C. Each well was washed and blocked overnight with 100 μL of 1% bovine serum albumin (BSA) in PBS. After washing, 100 μL of sample and broiler reference serum (RS10-102-3; Bethyl) were added and the plate was incubated for 1 h at 37°C. Thereafter, unbound material was washed away and 100 μL of HRP-conjugated goat anti-chicken IgA (A30-103P-35; Bethyl) in 1% BSA/PBS was added. After incubating for 1 h at 37°C, the plate was washed and 100 μL of TMB (CW0050; CWBIO, China) was added. The plate was then allowed to stand for 15 min at 37°C. After addition of 50 μL of 2.5 M H_2_SO_4_ to stop the reaction, absorbance at 450 nm was measured in a Titertek Multiskan Plus MKII plate reader.

### Plasma Metabolites

Plasma concentrations of triglyceride (TG), 5-HT, adiponectin (ADP), and non-esterified fatty acid (NEFA) were measured spectrophotometrically using commercial diagnostic kits (Jiancheng Bioengineering Institute, Nanjing, China). Plasma very-low density lipoprotein (VLDL) levels were measured as described by Griffin and Whitehead (1982).

The measurement of plasma tryptophan was measured as follows. Proteins were removed from plasma using 5-sulphosalicylic acid dihydrate (SSA, S3147; Sigma) before analyzing amino acid composition. The minimum purity was 99%, but the conditions used to remove the protein varied depending on the protein concentration of the sample. Briefly, 50 mg of pre-cooled SSA was added to each 1 mL of sample and mixed immediately. Then, the mixture was incubated at 4°C for 1 h and centrifuged at 15,000 *g* for 20 min (at 4°C). The supernatant was obtained and passed through a 0.2 μm filter. Finally, the supernatant was examined using an amino acid analyzer (L-8900; Hitachi, Japan).

### RNA Analysis

The expression of genes in the liver, abdominal fat and duodenum tissues was quantified using quantitative real-time polymerase chain reaction (qRT-PCR) with SYBR Green I labeling (04913914001, Roche, United States). The mRNA levels of the cytokines were only measured in duodenum according to the result of sIgA in intestinal content. Approximate 0.1 g tissue sample was homogenized with 10 times Trizol Reagent (15596-026, Invitrogen, San Diego, CA, United States). Total RNA was isolated by the guanidinium isothiocyanate method. The quality of RNA after DNase treatment was tested by electrophoresis on an agarose gel, and the quantity of RNA was determined using a biophotometer (Eppendorf, Germany). Then, 1 μg of total RNA was used for reverse transcription with a PrimeScript RT reagent kit (04897030001, Roche, United States) to prepare the cDNA. The primer sequences are shown in Table 2. Real-time PCR analysis was conducted using an Applied Biosystems 7500 Real-time PCR System (Applied Biosystems, Foster, CA, United States). Each RT-reaction served as the template in a 20 μL PCR reaction volume containing 0.2 μmol/L of each primer and SYBR Green I labeling (04913914001, Roche, United States). SYBR green fluorescence was detected at the end of each cycle to monitor the amount of PCR product. Real-time PCR was performed at 95°C for 10 s, followed by 40 cycles at 95°C for 5 s and 60°C for 40 s. SYBR green fluorescence was detected at the end of each cycle to monitor the amount of PCR product. A standard curve was plotted to calculate the efficiency of the real-time PCR primers. The gene-specific primers used for the analysis of chicken gene expression were listed in Table 2.

**TABLE 2:** Gene-specific primers used for the analysis of chicken gene expression.

Gene	GeneBank accession no.	Primer sequences (5′–3′)	Orientation	Product size (bp)
*ACC*	NM_205505	AATGGCAGCTTTGGAGGTGT TCTGTTTGGGTGGGAGGTG	Forward Reverse	136
*ADP*	NM_206991	ACCCAGACACAGATGACCGTT GAGCAAGAGCAGAGGTAGGAGT	Forward Reverse	239
*ADP-1R*	NM_001031027	GGAGAAGGTTGTGTTTGGGATGT TGGAGAGGTAGATGAGTCTTGGC	Forward Reverse	1355
*ADP-2R*	NM_001007854	ACACACAGAGACTGGCAACATC CCCAAGAAGAACAATCCAACAACC	Forward Reverse	779
*CPT1*	AY675193	GGAGAACCCAAGTGAAAGTAATGAA GAAACGACATAAAGGCAGAACAGA	Forward Reverse	135
*FAS*	J03860	TCCTTGGTGTTCGTGACG CGCAGTTTGTTGATGGTGAG	Forward Reverse	163
*PPAR*α	AY220305	CACCTACTACCGCAACAACG AGCAGATGAAGAAGACTCCCAG	Forward Reverse	218
*PPAR*γ	AF163809	AGACACCCTTTCACCAGCATCC AACCCTTACAACCTTCACAAGCA	Forward Reverse	167
*TBP*	NP_990434.1	ACAACAGCTTGCCGCCCTACG TTGCACCCTGAGGGGAGGCT	Forward Reverse	302
*IL-1*	NM_204524.1	CTCCTCCAGCCAGAAAGTGA GTAGCCCTTGATGCCCAGT	Forward Reverse	126
*IL-6*	HM179640.1	CGCCCAGAAATCCCTCCTC AGGCACTGAAACTCCTGGTC	Forward Reverse	152
*IL-8*	DQ393272.2	CTGCGGTGCCAGTGCATTAG AGCACACCTCTCTTCCATCC	Forward Reverse	139
*IL-10*	NM_001004414.2	CGCTGTCACCGCTTCTTCA TCCCGTTCTCATCCATCTTCTC	Forward Reverse	88
*IgA*	S40610.1	AGGGCAATGAGTTCGTCTGT AGGAGGGTCACTTTGGAGGT	Forward Reverse	112
*TGF-*β	XM_015284275	AGGATCTGCAGTGGAAGTGGAT CCCCGGGTTGTGTTGGT	Forward Reverse	187
*TNF-*α	HQ739087.1	GAGCGTTGACTTGGCTGTC GCTGCACATACACAGTCTGA	Forward Reverse	129
*5-HT2A*	XM425628.4	AAAGTCCGCCGTGCTACTC AGAGGCCACTTGTATCCGTA	Forward Reverse	250
β*-actin*	NM_205518.1	CTGGCACCTAGCACAATGAA CTGCTTGCTGATCCACATCT	Forward Reverse	123

The relative amount of mRNA of a gene was calculated according to the method of Livak and Schmittgen (2001). The mRNA levels of these genes were normalized to β-actin levels (ΔCT). The ΔCT was calibrated against an average of the control chickens. The linear amount of target molecules relative to the calibrator was calculated by 2^–ΔΔCT^. Therefore, all gene transcription results are reported as the *n*-fold difference relative to the calibrator. The specificity of the amplification product was verified by the standard curve and dissolution curve.

### Western Blot Analysis

Duodenum and abdominal fat tissue samples were homogenized in 1 mL of lysis buffer (Beyotime, Jiangsu, CN) and centrifuged at 12,000 *g* for 10 min at 4°C. The supernatant was collected. Next, total proteins were extracted and the concentration was measured with a BCA Protein Assay Kit (Beyotime, Jiangsu, China). For western blot analysis, equal amounts of protein (duodenum tissue: 50 μg; abdominal fat tissue: 30 μg) were denatured for 10 min at 100°C and then separated in 7.5 and 12% sodium dodecyl sulfate (SDS)-polyacrylamide gels. Proteins were transferred to polyvinylidene fluoride (PVDF) microporous membranes (Millipore) at 200 mA at 4°C for 2 h. After 1 h in blocking solution (Beyotime) at room temperature, the membranes were incubated overnight at 4°C with primary antibodies specific for ADP (aa32–244; LsBio #LS-C292655) and ADP1R (mAbcam50675; Abcam #ab50675), rabbit monoclonal antibodies (mAbs) specific for phospho-NF-κB (NF-κB) p65 (Ser536) (93H1), phosphor-p38 MAPK (Thr180/Tyr182), phospho-p70S6 Kinase (p70S6K) (Thr389) (108D2), phospho-mTOR (Ser2448), total NF-κB p65 (C22B4), p38 MAPK, p70S6K (49D7), and mTOR (all from Cell Signaling Technology, Inc.), and mouse mAbs specific for tubulin (AT819; Beyotime) and β-actin (AA128; Beyotime). The blots were then incubated for 4 h at 4°C with anti-rabbit or anti-mouse IgG-conjugated horseradish peroxidase antibodies (Beyotime), and binding was detected by ECL (Beyotime). Bands were visualized and analyzed using a FUSION device.

### Statistical Analysis

Prior to analysis, all data were examined for the homogeneity and normal distribution plots of variances among the treatments by using UNIVARIATE procedure. For variables feed intake, BW gain, and FCR, a one-way ANOVA model was used to estimate the main effects of 5-HTP treatment on a per-pen basis (SAS version 8e, SAS Institute, 1998). For the variables gene expression and plasma metabolite, a one-way ANOVA model was used to estimate the main effect of feeding treatment for individual broiler. For the data of LPS exposure, a two-way ANOVA model was used to estimate the main effects of 5-HTP and LPS treatment, as well as their interactions for individual chickens. When the main effect of the interaction was significant, multiple comparisons were conducted by Duncan’s multiple range analysis. *P* < 0.05 was considered statistically significant.

## Results

### Growth Performance, Organ Index, and Plasma Metabolites

The average daily feed intake was not significant influenced (*P* > 0.05) by 5-HTP treatment in the periods of 8–21 days and 22–35 days of age. In contrast, the feed intake during the entire experiment period was decreased by 5-HTP treatment (*P* < 0.05). 5-HTP treatment led to reduced BW gain in the period of 8–21 days of age (*P* = 0.050) and a trend toward reduced BW gain (*P* = 0.056) from 8 to 35 days of age, but had no significant influence in the period of 22–35 days of age (*P* > 0.05, Table 3). FCR was not changed by 5-HTP treatment (*P* > 0.05).

**TABLE 3:** Effect of dietary 5-hydroxytryptophan (0.2%, 5-HTP) on growth performance of broilers.

	Control	5-HTP	Significance
Initial body weight at 7-day age, g	141.2 ± 5.37	139.0 ± 6.61	NS
**Feed intake, g/d**			
8–21 days	55.8 ± 0.92	52.5 ± 1.64	NS
22–35 days	128.5 ± 1.66	124.5 ± 2.87	NS
8–35 days	92.2 ± 0.73^a^	88.5 ± 1.25^b^	0.033
**Body weight gain, g/d**			
8–21 days	39.9 ± 1.23	36.3 ± 0.64	0.050
22–35 days	75.9 ± 1.55	73.7 ± 1.58	NS
8–35 days	57.9 ± 0.80	55.0 ± 1.02	0.057
**FCR, g/g**			
8–21 days	1.40 ± 0.02	1.44 ± 0.02	NS
22–35 days	1.70 ± 0.04	1.69 ± 0.05	NS
8–35 days	1.59 ± 0.02	1.61 ± 0.03	NS

*Values were presented as Mean ± SEM (n = 4); ^*a,b*^P < 0.05; NS, not significant.*

**FIGURE 1:**
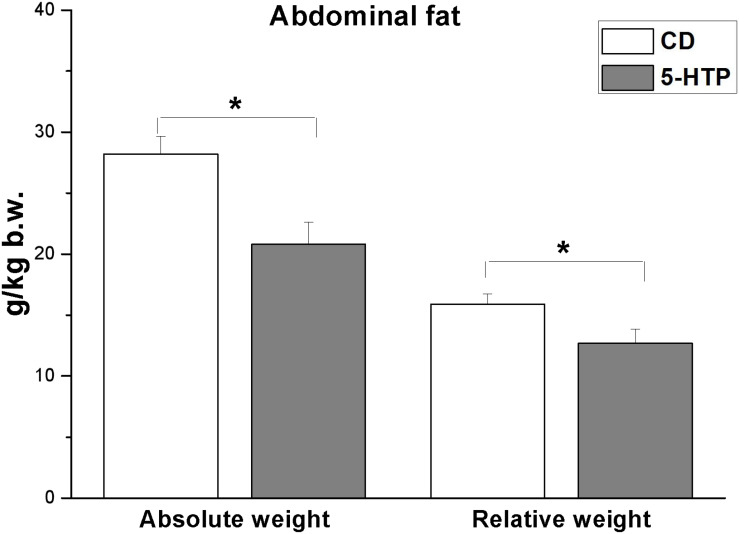
Effect of dietary 5-hydroxytryptophan supplementation (0.2%, T-HTP) on the abdominal fat pad of broilers. CD, control group. Data are presented as mean ± SD (*n* = 8); *P < 0.05.

In addition, there was a reduction in abdominal fat weight (Figure 1; *P* < 0.05) but no significant change in liver weight (*P* > 0.05, data not shown). There was no significant difference (*P* > 0.05) in plasma TG, 5-HT, VLDL, ADP, NEFA, and tryptophan levels between 5-HTP broilers and control ones (Table 4).

**TABLE 4:** Effect of dietary 5-hydroxytryptophan treatment (0.2%, 5-HTP) on plasma concentrations of tryptophan, triglyceride (TG), very-low density lipoprotein (VLDL), non-esterified fatty acid (NEFA), adiponectin (ADP), and 5-hydroxytryptamine (5-HT).

	Control	5-HTP	Significance
TG, mmol/L	0.27 ± 0.04	0.26 ± 0.03	NS
VLDL, Abs*1000	12.4 ± 2.20	13.8 ± 1.75	NS
NEFA, mmol/L	1.82 ± 0.12	1.84 ± 0.08	NS
ADP, ng/mL	3.07 ± 0.07	3.24 ± 0.30	NS
5-HT, ng/mL	43.8 ± 3.30	45.6 ± 2.53	NS
Tryptophan, g/L	9.13 ± 0.37	9.44 ± 0.34	NS

**Data were presented as Mean ± SD (n = 8); NS: P > 0.05.**

### Expression of Genes Associated With Lipid Metabolism in Liver and Abdominal Fat Pad

**FIGURE 2:**
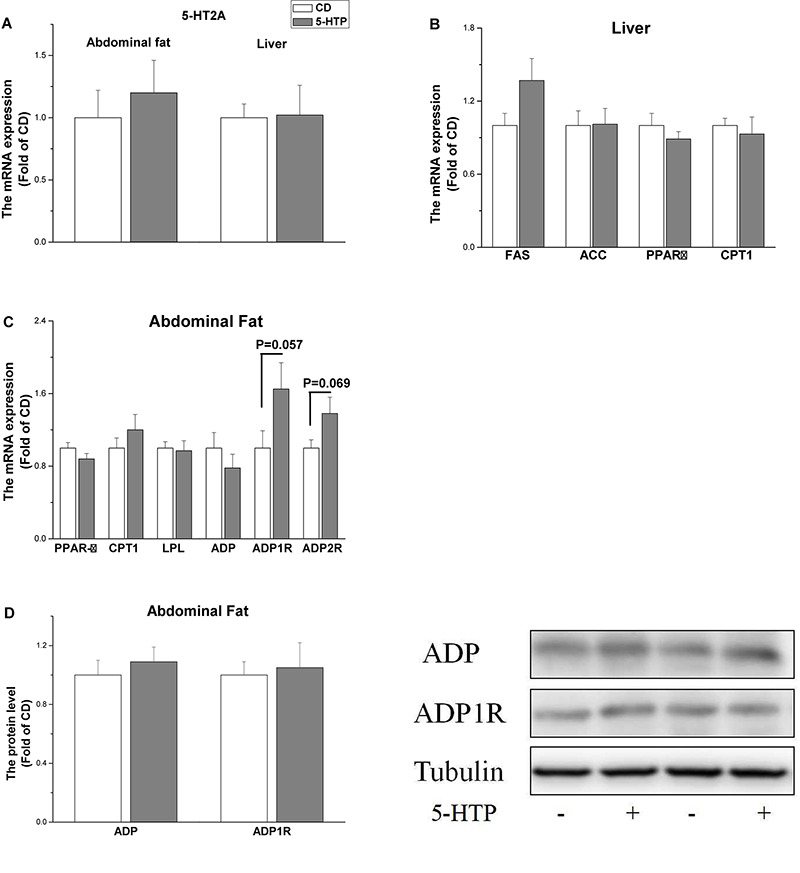
Effect of dietary 5-hydroxytryptophan supplementation (0.2%, 5-HTP) on the expression level of genes related to lipid metabolism in the liver and abdominal fat pad **(A)** serotonin receptor 2A (5-HT2A) in the liver and abdominal fat; **(B)** fatty acid synthase (FAS), acetyl-CoA carboxylase (ACC), carnitine palmitoyltransferase 1 (CPT1), and peroxisome proliferator-activated receptor (PPAR)α in the liver; and **(C)** PPARγ, CPT1, lipoprotein lipase (LPL), adiponectin (ADP), and ADP1R and ADP2R in abdominal fat; **(D)** ADP and ADP1R proteins in abdominal fat. Data are presented as the mean ± SD (*n* = 6).

5-HTP treatment had no significant effect (*P* > 0.05) on the mRNA level of 5-hydroxytryptamine receptor 2A (5-HT2A) in the liver and abdominal fat pad (Figure 2A). The mRNA level of fatty acid synthase (FAS), acetyl-CoA carboxylase (ACC), carnitine palmitoyltransferase 1 (CPT1), and peroxisome proliferator-activated receptor α (PPAR α) in the liver was unchanged by 5-HTP treatment (*P* > 0.05, Figure 2B). In abdominal fat, the mRNA levels of PPARγ, lipoprotein lipase (LPL), CPT1, and ADP were not significantly influenced by 5-HTP treatment (*P* > 0.05, Figures 2B,C). By contrast, the mRNA level of adiponectin receptor 1 (ADP1R) (*P* = 0.057) and ADP2R (*P* = 0.069) tended to be increased in 5-HTP-treated chickens compared with control (Figure 2C). The protein expression levels of ADP and ADP1R, however, were not changed by 5-HTP treatment (*P* > 0.05; Figure 2D).

### Concentration of sIgA in Serum and Intestinal Contents

**FIGURE 3:**
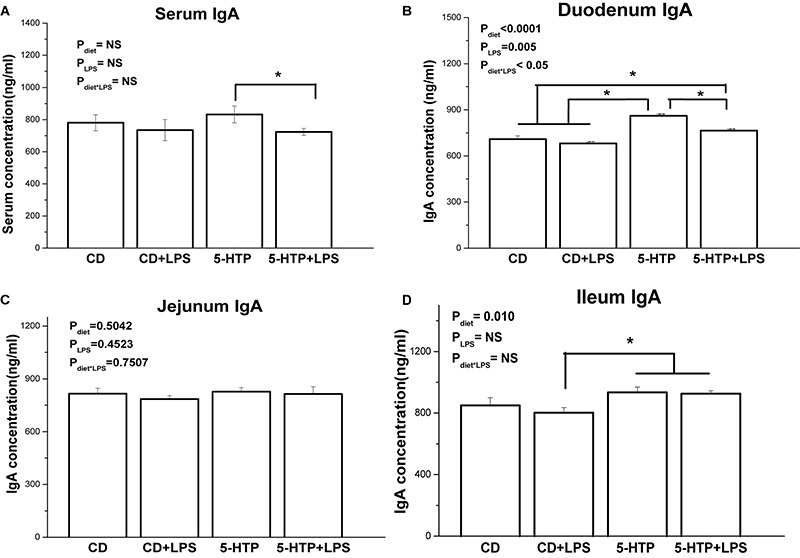
Effect of dietary 5-hydroxytryptophan supplementation (0.2%, 5-HTP) and lipopolysaccharide (LPS, 1 mg/kg of body weight) on secretory immunoglobulin A (sIgA) concentrations in serum and intestinal contents. **(A)** serum sIgA; **(B)** sIgA in duodenum contents; **(C)** sIgA in jejunum contents; and **(D)** sIgA in ileum contents. Data are presented as the mean ± SD (*n* = 8); *P < 0.05.

The IgA concentration in serum from the 5-HTP group fell after LPS treatment (*P* < 0.05; Figure 3A), whereas LPS had no detectable effect (*P* > 0.05) in CD chickens. Duodenal contents from 5-HTP chickens showed significantly higher sIgA concentrations than those from CD chickens (*P* < 0.05); however, LPS treatment reduced the sIgA concentration in the duodenum of 5-HTP chickens but not in that of CD chickens (*P* < 0.05; Figure 3B). By contrast, the IgA concentration in jejunal contents was unaffected by LPS or diet (*P* > 0.05; Figure 3C). CD-LPS chickens showed lower sIgA levels in the ileum contents than 5-HTP chickens (*P* < 0.05; Figure 3D).

### Expression of Cytokine-Encoding mRNA in Duodenum

**FIGURE 4:**
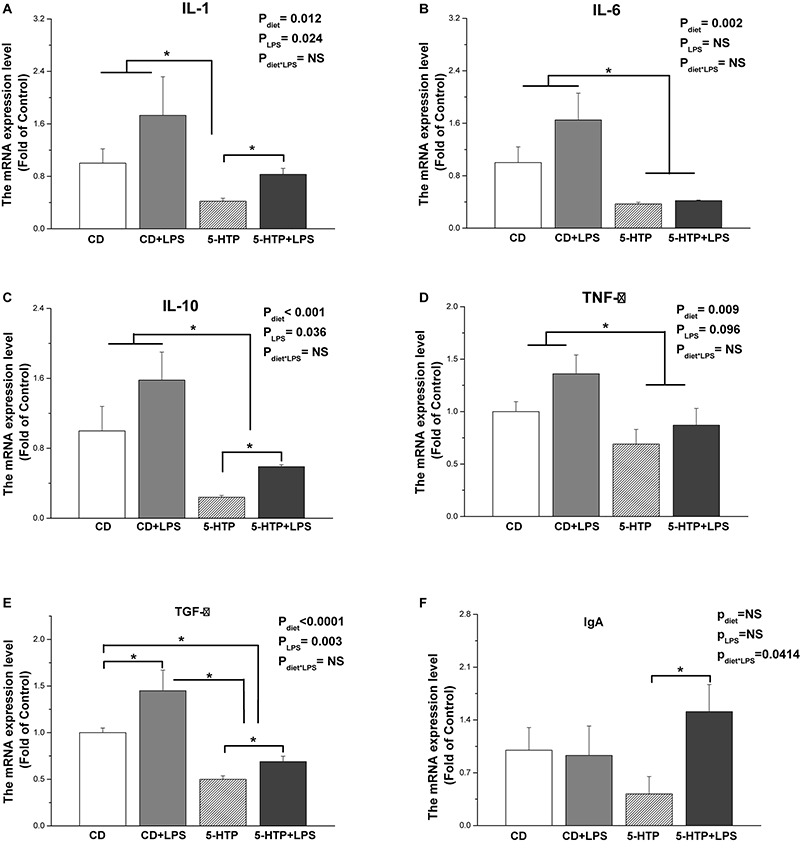
Effect of dietary 5-hydroxytryptophan supplementation (0.2%, 5-HTP) and lipopolysaccharide (LPS, 1 mg/kg of body weight) on cytokine mRNA levels in the duodenum. **(A)** Interleukin (IL)-1; **(B)** IL-6; **(C)** IL-10; **(D)** transforming growth factor-β (TGF-β); **(E)** tumor necrosis factor-α (TNF-α); and **(F)** IgA. CD, control group; data are presented as the mean ± SD (*n* = 8); *P < 0.05.

Treatment with 5-HTP decreased expression of mRNA encoding interleukin 1 (*IL-1*, *P* < 0.05), *IL-6* (*P* < 0.01), *IL-10* (*P* < 0.001), tumor necrosis factor α (*TNF-*α) (*P* < 0.01), and transforming growth factor β (*TGF-*β, *P* < 0.001) (Figures 4A–E). Treatment with 5-HTP and LPS had a significant effect (*P* < 0.05) on *IgA* mRNA levels: *IgA* mRNA levels in the 5-HTP group (*P* < 0.05), but not in the CD group (*P* < 0.05), were increased by LPS (Figure 4F). LPS treatment led to significant upregulation of *IL-1* (*P* < 0.05), *IL-10* (*P* < 0.05), and *TGF-*β (*P* < 0.01) mRNA. However, LPS tended to downregulate *TNF-*α mRNA levels (*P* = 0.098). *IL-6* mRNA levels were not affected by LPS (*P* > 0.05).

### Phosphorylation of mTOR, MAPK, and NF-κB in Duodenum

**FIGURE 5:**
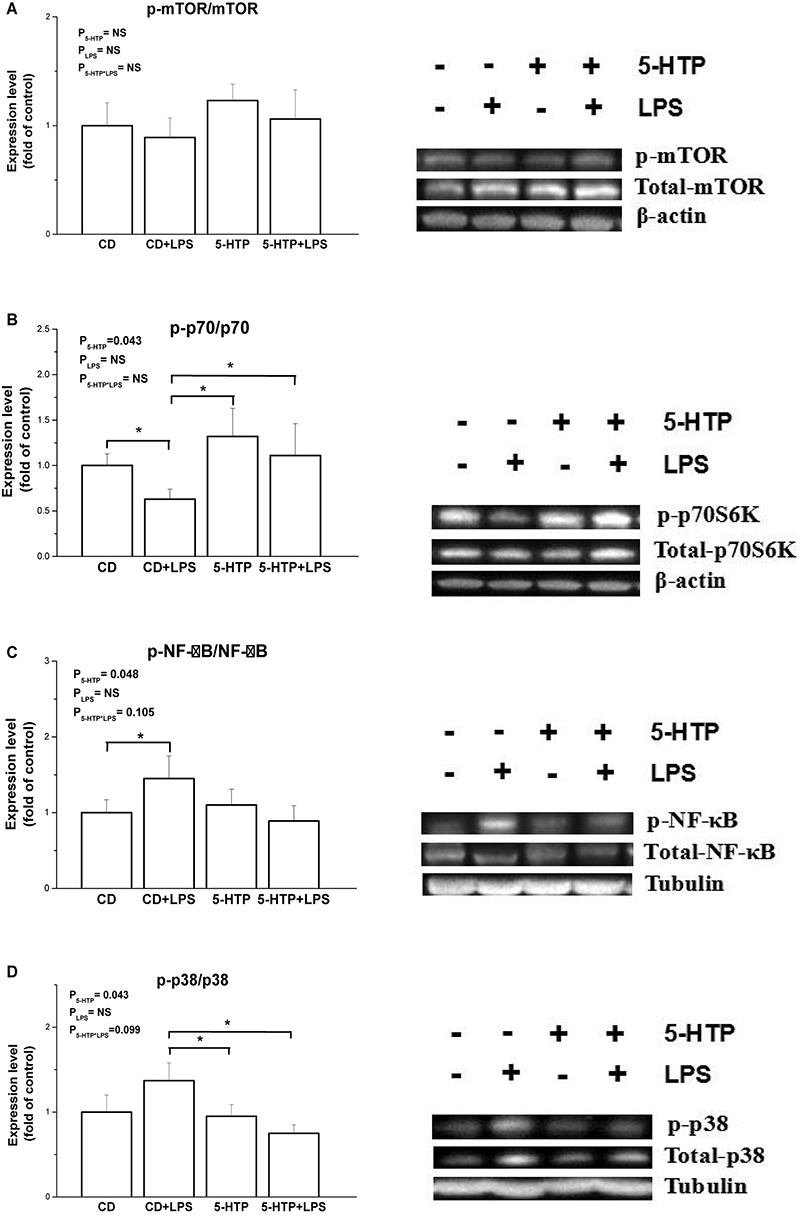
Effect of dietary 5-hydroxytryptophan supplementation (0.2%, 5-HTP) and lipopolysaccharide (LPS) (intraperitoneal injection at a dose of 1 mg/kg of body weight) on phosphorylation of signal molecules. **(A)** Ribosomal p70S6 kinase (p70S6K), **(B)** mammalian target of rapamycin (mTOR), **(C)** nuclear factor-kappa B (NF-κB) p65, and **(D)** p38 mitogen-activated protein kinase (MAPK), in the duodenum. CD, control group; data are presented as the mean ± SD (*n* = 8); *P < 0.05.

Compared with the CD group, treatment with 5-HTP increased phosphorylation of ribosomal p70S6K (*P* < 0.05, Figure 5A), but suppressed phosphorylation of nuclear factor-kappa B (NF-κB) (*P* < 0.05; Figure 5C) and mitogen-activated protein kinase (p38 MAPK) (*P* < 0.05; Figure 5D). By contrast, there were no differences between the control birds and birds treated with LPS. Neither LPS nor 5-HTP had a significant effect on phosphorylation of mTOR (*P* > 0.05; Figure 5B).

## Discussion

### 5-HTP Suppresses Feed Intake

In the present study, 5-HTP was supplemented in the broiler diet for 28 days and the zootechnical performance was measured at 35 days of age, which is in the period of maximum growth rate of body weight body of modern strain of broilers (Scheuermann et al., 2003). The results presented herein show that administration of 5-HTP reduced both FI and BW gain in broilers. The lack of change in the FCR indicated that the reduction in BW gain was caused mainly by the reduced FI. It is interesting to note that the variation of feed intake and body weight gain was greater in 5-HTP treatment, compared with that of control. As the variation of FCR in 5-HTP group was comparable to control group, the variation in body weight gain seems to be a result of feed intake. 5-HT has an inhibitory effect on appetite in mammals (Brewerton, 1995) and avian species (Steffens et al., 1997). In human, increased activity of the 5-HT system suppresses feeding behavior (Brewerton, 1995). 5-HT activates pro-opiomelanocortin neurons via the 5-HT2C receptor while inhibiting neuropeptide Y/agouti-related protein neurons via the 5-HT1B receptor (Heisler et al., 2002). In chicken, ICV injection of serotonin decreased food intake (Zendehdel et al., 2017). Central 5-HT is associated with feeding/foraging behavior of chicken (de Haas and van der Eijk, 2018). Hence, the present result suggests that 5-HTP administration suppresses appetite and reduces feed intake of broilers via the serotonergic system.

### 5-HTP Reduces Abdominal Fat Accumulation

Accumulating evidence suggests that peripheral 5-HT may affect the energy homeostasis (Tecott, 2007). The present results show that dietary treatment with 5-HTP inhibits accumulation of abdominal fat without affecting the level of tryptophan in plasma, indicating that the change is induced mainly by 5-HTP. In mammal, peripherally, 5-HT plays a role in development of obesity (Haub et al., 2011; Kim et al., 2011). Pharmacological inhibition of 5-HT synthesis or TPH1 knockout in adipose tissues leads to inhibition of lipogenesis in epididymal white adipose tissue, suggesting 5-HT has localized effects on adipose tissues (Oh et al., 2015). The present result indicates that 5-HT plays a different effect from that of mammal on lipid metabolism in chicken. *De novo* lipogenesis in birds, as well as in humans, occurs mainly (although not exclusively) in the liver (Bedu et al., 2002). Therefore, we investigated expression of genes related to lipogenesis in both liver and abdominal fat. In the liver, expression of mRNA encoding FAS and ACC was unaltered by 5-HTP treatment, indicating that hepatic lipogenesis was not affected. In abdominal fat, LPL and PPARγ play roles in lipid deposition and adipocyte differentiation (Schoonjans et al., 1996; Wang and Eckel, 2009). The unchanged expression of PPARγ and LPL mRNA in 5-HTP chickens indicates that adipocyte development is not affected by 5-HTP treatment. The mRNA expression of gene encoding 5-HT2A in both liver and abdominal fat was consistent with the finding of unaffected circulating levels of 5-HT. In rats, however, the endogenous serotonin levels increased in the plasma and brain at 4 h after 5-HTP administrations (Sharma et al., 2019).

We further investigated expression of two genes related to lipid oxidation: CPT1 and PPARα (Preidis et al., 2017). Transcription of PPARα and CPT1 in liver was unchanged by 5-HTP, suggesting that 5-HTP plays little if any role in lipid utilization by the liver. Adipose tissue affects energy homeostasis by ADP, which promotes fatty acid oxidation and glucose utilization (Combs et al., 2001; Zhou et al., 2005; Liu et al., 2012). ADPR agonists and ADP sensitizers may serve as versatile treatment strategies for obesity-linked diseases such as metabolic syndrome and diabetes (Kadowaki and Yamauchi, 2005). The chickens with a high pecking tendency have low peripheral 5-HT (de Haas and van der Eijk, 2018). Moreover, the locomotor activity is higher in the line selected for high levels of feather pecking compared to control and the line selected for low levels of feather pecking (Kjaer, 2009). The acutely enhanced 5-HT level decreases motor activities of chicks (Matsunami et al., 2012). Hence, the involvement of locomotion activity on the energy expenditure remains to be elucidated. Here, we found that neither circulating ADP levels nor ADP1R and ADP2R mRNA and ADP1R protein levels in abdominal fat were altered by 5-HTP treatment, suggesting that ADP may not be a target of 5-HTP. Collectively, the present result suggests that the reduced feed intake should be responsible for the suppressed abdominal fat accumulation by 5-HTP treatment. Moreover, the effect of 5-HTP on lipid utilization should be investigated further.

The reduced abdominal fat deposit is accompanied with the increased muscle fat accumulation and improved polyunsaturated fatty acid (PUFA) contents (Saleh et al., 2012, 2014). Moreover, the 5-HT receptor signaling could be influenced by the lipid microdomains (lipid raft) in plasma membrane via the regulatory effect on G-proteins and meanwhile, the lipid raft molecular organization and raft-associated protein distribution are highly susceptible to modulation by long chain *n*−3 PUFAs (Liu S. Q. et al., 2018). Hence, the effect of 5-HTP on the fat accumulation and the fatty acid profile in muscle needs to be investigated further.

### 5-HTP Is Beneficial to the Intestinal Immune Function With the Involvement of mTOR/p70S6K Signaling Pathway

5-HT is produced by enterochromaffin cells and can regulate gut physiology by promoting intestinal inflammation (Margolis et al., 2014). The 5-HT type 7 receptor, one of the most recently identified members of the 5-HT receptor family, plays a critical role in regulating mucosal inflammation and immune responses (Kim et al., 2013). Therefore, we examined the effect of relative long-term 5-HTP supplementation (4 weeks) on LPS-induced gut inflammation in chickens.

sIgA, the most abundant immunoglobulin in mucosal secretions, plays an important role in the intestinal mucosal immune system and in maintenance of mucosal homeostasis. In chickens, IgA secreted by the intestine is similar to mammalian sIgA and is associated with a protein homologous to a mammalian secretory component of 350,000 Daltons (Watanabe et al., 1975). The increased sIgA concentration in the duodenum and ileum of 5-HTP chickens indicates that 5-HTP treatment may increase secretion of sIgA into the intestinal tract. Furthermore, 5-HTP treatment decreased the mRNA levels of IL-1, IL-6, IL-10, TNF-α, and TGF-β in the duodenum. This result is in contrast to the observations in mammals. Ghia et al. (2009) found that 5-HT stimulated production of IL-1 and IL-6 by peritoneal resident macrophages. Direct exposure of colon epithelial cells to 5-HT triggers inflammatory reactions (Regmi et al., 2014). After LPS administration, however, we found that 5-HTP supplementation increased serum and duodenal sIgA concentrations, in contrast to the lack of response noted in CD chickens. This result was in accordance with the significant interaction between 5-HTP and LPS treatments on duodenum IgA mRNA level, suggesting that 5-HTP improves IgA secretion upon to LPS challenge. As the interaction of 5-HTP and LPS was only observed in duodenum in the present study, we further determined the transcriptional level of relevant cytokines. LPS treatment significantly changed the expression level of IL-1, IL-10, and TGF-β, suggesting that LPS induces inflammation response. The lack of interaction of LPS and 5-HTP on all the expression of measured genes, however, indicated that 5-HTP had no regulation on LPS-induced inflammation at the present condition. Hence, the effect of 5-HTP on LPS-induced inflammation response needs to be investigated further.

mTOR, a highly conserved and ubiquitously expressed signaling molecule, plays roles in cell growth and proliferation and other cellular functions. mTOR senses metabolic signals and exerts anabolic effects by stimulating protein synthesis and ribosomal biogenesis, thereby increasing cell proliferation and promoting cell survival (Wang et al., 2011; Fielhaber et al., 2012). mTOR exerts its regulatory effects by stimulating phosphorylation of the downstream target protein p70S6K. 5-HT activates the mTOR pathway in mammals; for example, exposure to 5-HT activates the hepatic mTOR/p70S6K pathway in rat liver (Li et al., 2016). Our previous study indicated that the mTOR pathway is associated with intestinal immunity (Liu et al., 2016; Liu J. J. et al., 2018). Our present data show that the 5-HTP diet restored the phosphorylation level of p70S6K, which was inhibited by LPS, indicating that the p70S6K signaling pathway may play a role in the regulatory effects of 5-HTP on intestinal mucosal immunity in chickens.

In LPS-stimulated murine macrophages, cross-talk between the mTOR and NF-κB pathways plays a role in LPS-induced responses (Dos Santos et al., 2007). The Akt/mTOR cascade differentially modulates LPS-induced cytokine production by peripheral blood mononuclear cells (Schaeffer et al., 2011). Blocking the mTOR pathway in human monocytes suppresses expression of chemokines by modulating the MAPK and NF-κB p65-mediated signaling pathways (Lin et al., 2014). Here, we found that phosphorylation of NF-κB and p38 MAPK in 5-HTP-treated chickens was inhibited while p-p70 was stimulated, regardless of LPS administration. Adversely, exposure to 5-HT induces inflammatory reactions in colon epithelial cells and the underlying mechanism is associated with Nox2-activated signaling pathways, including ERK/p38 MAPK and NF-κB (Regmi et al., 2014). Hence, the pathway of 5-HTP regulating intestinal immune function remains to be elucidated. In line with the result of cytokine gene expression, no detectable interaction of 5-HTP and LPS was observed. In the present study, LPS had no significant influence on the phosphorylation levels of mTOR, p70, NF-κB, and p38 MAPK. However, the increased phosphorylation of NF-κB was detected in control chickens in response to LPS, suggesting that the activated NF-κB/p38 pathway by LPS treatment cannot be excluded, as the phosphorylation may occur quickly (Liu et al., 2016; Liu J. J. et al., 2018). Collectively, the results suggest that 5-HTP is beneficial to intestinal immune function with the involvement of mTOR/p70S6K and MAPK/NF-κB pathway.

## Conclusion

Taken together, the results presented herein suggest that dietary supplementation with 5-HTP reduces feed intake and the accumulation of abdominal fat and is beneficial to the intestinal immune function.

## Data Availability Statement

The sequencing data has been deposited into Figshare (10.6084/m9.figshare.2291212).

## Ethics Statement

The animal study was reviewed and approved by the Animal Care and Use Committee of Shandong Agricultural University, the Guidelines for Experimental Animals established by the Ministry of Science and Technology.

## Author Contributions

HW, SL, and HL conceived and designed the experiments and wrote the manuscript. HW and SL performed the experiments and analyzed the data. JL and LW conducted some measurements. XW, JZ, and HJ provided essential reagents. All authors read and approved the final manuscript.

## Conflict of Interest

The authors declare that the research was conducted in the absence of any commercial or financial relationships that could be construed as a potential conflict of interest.
